# Access to primary healthcare services for the Roma population in Serbia: a secondary data analysis

**DOI:** 10.1186/1472-698X-11-10

**Published:** 2011-08-18

**Authors:** Leanne Idzerda, Orvill Adams, Jonathan Patrick, Ted Schrecker, Peter Tugwell

**Affiliations:** 1 Institute of Population Health, University of Ottawa, Ottawa, Canada; 2 Director of Orvill Adams & Associates Incorporated, Health Systems Policy and Workforce Planning, Ottawa, Ontario, Canada; 3 Telfer School of Management, University of Ottawa, Ottawa, Canada; 4 Department of Medicine, University of Ottawa, Ottawa, Canada

## Abstract

**Background:**

Serbia has proclaimed access to healthcare as a human right. In a context wherein the Roma population are disadvantaged, the aim of this study was to assess whether the Roma population are able to effectively access primary care services, and if not, what barriers prevent them from doing so. The history of the Roma in Serbia is described in detail so as to provide a context for their current vulnerable position.

**Methods:**

Disaggregated data were analyzed from three population groups in Serbia; the general population, the Roma population, and the poorest quintile of the general population not including the Roma. The effective coverage framework, which incorporates availability, affordability, accessibility, acceptability, and effectiveness of health services, was used to structure the secondary data analysis. Acute respiratory infection (ARI) in children less than five years of age was used as an example as this is the leading cause of death in children under 5 years old in Serbia.

**Results:**

Roma children were significantly more likely to experience an ARI than either the general population or the poorest quintile of the general population, not including the Roma. All three population groups were equally likely to not receive the correct treatment regime of antibiotics. An analysis of the factors that affect quality of access to health services reveal that personal documentation is a statistically significant problem; availability of health services is not an issue that disproportionately affects the Roma; however the geographical accessibility and affordability are substantive issues that disproportionately affect the Roma population. Affordability of services affected the Roma and the poorest quintile and affordability of medications significantly affected all three population groups. With regards to acceptability, mothers from all three population groups are equally likely to recognize the importance of seeking treatment.

**Conclusions:**

The Roma should be assisted in applying for personal documentation, the geographical accessibility of clinics needs to be addressed, and the costs of healthcare visits and medications should be reviewed. Areas for improvement specific to ARI are the costs of antibiotics and the diagnostic accuracy of providers. A range of policy recommendations are outlined.

## 1.0 Background

As with many other countries undergoing transitions from authoritarian rule to democracy, Serbia has incorporated health as a human right in its Constitution [1]. In 2005, the Government of Serbia adopted a set of health related laws specifying that health services should be physically, economically, and geographically accessible [2]; in addition, patients have the right to access health services without discrimination [3]. Previous studies [4,5] indicate that the Roma population are one of the largest vulnerable groups in Serbia and that they may be disadvantaged across a number of dimensions including place of residence, ethnicity, occupation, gender, religion, education level, and socioeconomic status [6]. Although the Roma have a right to primary healthcare services, defined as an accessible health system wherein services are delivered to a community by an accountable healthcare professional [7], it is not clear whether they are in fact able to access these services effectively. This research attempts to address that question.

In this paper, we first describe the situation of the Roma in Serbia including their history, current living conditions, and vulnerability to ill health described in terms of both the social determinants of health and individual factors. We then assess whether the Roma, as a vulnerable population, are able to effectively access primary care services, and if not, what barriers prevent them from doing so. We use the example of acute respiratory infection in children under the age of 5 years to demonstrate where the gaps in coverage exist as this is the leading cause of death among children of this age group in Serbia. We conclude with some recommendations for policy change, informed by considerations of equity and by recent research on health systems and the right to health.

### 1.1 The Roma population in Serbia

Approximately 5.2 million Roma live in Central and Eastern Europe [8], with over 108 000 people defining themselves as Roma in Serbia [9]. It is thought that the actual number of Roma people living in Serbia is four to five times higher than this [10]. Many, however, may not declare themselves as Roma due to widespread discrimination. Due to their marginalized position in society, the Roma have largely been subject to description and stereotyping by the majority population based on their physical features and socioeconomic status [11,12].

### 1.2 Subgroups of Roma in Serbia

The Roma in Serbia may be divided into three main groups based on their personal histories: the domestic Roma, internally displaced persons (IDP) from Kosovo, and returnees from Western Europe.

*The domestic Roma *are those who have lived in Serbia their entire lives. According to the UNDP vulnerability survey, 98% of the domestic Roma population had lived in the same location for the past 15 years, compared to 90% of the non-Roma population interviewed [13] For the most part, domestic Roma communities live in settlements on the outskirts of cities or in smaller industrial towns.

*Large numbers of Internally Displaced Roma (IDPs) from Kosovo *are a result of mass migrations between 1991 and 1995 due to great instability in that region. A second wave of IDPs fled to Serbia because of the resurgence of violence in the area in 1999 [14]. In 2007, there were 22,457 Roma IDPs registered. However, the United Nations Development Program estimates that the real number of IDP Roma is probably closer to 50,000 [13]. While a small number of IDPs end up in government-run or "unofficial" collective centers, the majority of Roma IDPs find accommodation in Roma settlements [14]. *Returnees from Western Europe *are those people for whom it is mandatory to return to Serbia following failed attempts at asylum seeking. The number of Roma who have already returned, or who are awaiting return, is not available; however, according to the Council of Europe, it is estimated that up to 100,000 people will be returned to Serbia with the majority coming from Germany, Sweden, France, and Switzerland. Most of the returnees are Roma, with estimates ranging from 60 to 75% of the total returnee population [15]. Upon return, the Roma population often moves into temporary housing in Roma settlements that are close to major city centers.

### 1.3 Defining the Roma Population

From this assessment, one can see that the Roma population in Serbia is not one homogenous population group, but rather a collection of subgroups with different life histories; thus defining the Roma population in this study is quite difficult. In addition, some have argued that defining a group based on ethnicity may cause further discrimination [13]. Defining the Roma based on self identification may mitigate this problem, however many Roma may not define themselves as Roma due to fear of discrimination. In order to overcome these problems, this research focused on communities with higher concentrations of persons at risk; i.e. those people living in settlements. It is assumed, based on previous research and government sources, that the vast majority of persons living in these settlements are in fact Roma [4].

#### 1.3.1. Location and Size of Roma settlements

The Roma settlements are distributed across Serbia with the majority of Roma living in Southern Serbia and near large urban centers. The Roma settlements in Serbia range in size from small settlements containing only a few households to some settlements containing over 5000 residents. The types of settlements also vary and include: old rural villages, settlements on the outskirts of cities, and inner city slums. Living conditions within settlements vary considerably, ranging from extremely poor slum housing, such as tin shacks or cardboard houses, to well-maintained brick houses [16].

#### 1.3.2 Family structure within Roma settlements

Defining family structure and social roles is important as these allow us to better understand the social dynamics of households. Households in Roma settlements tend to be multigenerational with the typical household consisting of at least one grandparent, usually the grandmother, their sons and their wives, and grandchildren. Social roles in Roma households are very well-defined. The grandparent is the head of the household and controls all resources, including economic resources, which are handed to them by the sons [17]. Boys and men are expected to work and support their families from a very young age regardless of their marital status. Typical employment includes factory work and labour intensive jobs, such as garbage collection. A large percentage of Roma also collect second-hand materials from dumps and sell these to recycling plants [10,13].

Women are expected to marry and take care of the children and the household [17]. Following marriage, the girl will move in with her husband's family and often occupies the lowest rung on the social hierarchy until she bears children; thus, fertility and childbirth are crucial to her social role. Because of these expectations, the average age of marriage for a girl is 17 years old, with 12% of girls marrying before 15 years of age [17,18]. Childbirth is usually one and a half years after marriage. The Roma tend to have very large households with women bearing an average of 3.3 children [17] compared to 1.38 children in the general population [19]. Due to the larger number of children as well as the multigenerational structure of households, Roma households are significantly larger than those in the general population.

#### 1.3.3 Roma as a disadvantaged population

One way to assess whether the Roma are disadvantaged is using an equity lens and the social determinants of health framework. Equity is different from inequality. Inequality is defined as a difference between population groups, regardless of whether this difference is fair. Equity has a moral dimension and is deemed present if (1) a difference or inequality exists, (2) the difference is unfair or unjust, and (3) the difference is avoidable or remediable [20].

The Commission on the Social Determinants of Health (CSDH) attempts to address inequity by looking at the larger societal picture and asking, "What are the causes of the causes?" In other words, what societal factors affect health? The CSDH framework considers health inequities as a "result of a complex system operating at global, national, and local levels which shapes the way society, at national and local levels, organizes its affairs and embodies different forms of social position and hierarchy." [21]. The CSDH report outlines five important areas in which inequities should be addressed: early child development; housing; fair employment; social protection; and universal healthcare [22]. Data on the indicators of inequity identified by the CSDH are summarized in table 1. This table compares the general population, the poorest quintile (not including the Roma population), and those living in Roma settlements.

**Table 1:** Comparison of Roma and non-Roma Health Status Indicators

Health Status Indicator	General Population	Poorest Quintile (20%)	Roma
**EARLY CHILDHOOD DEVELOPMENT**			

Low Birth Weight (< 2500 g)	4.9%	8.6%	9.3%
Prevalence of Stunting (moderate and severe)	5.4%	9.0%	20.0%
Enrolled in Primary School	99%	95.6%	73.6%
Complete Primary School	76.9%	60.9%	27.2%

**HOUSING**			
Electricity Supply	99.9%	99.2%	96.9%
Water Supply	97.6%	82.3%	72.9%
Sewerage System	96.0%	72.5%	59.4%

**EMPLOYMENT**			
Formally employed*	26.6%	13.9%	4.8%
Unemployed - seeking employment *	15.3%	11.3%	31.9%
Independent Agricultural Worker	5.9%	10.4%	4.4%

**SOCIAL PROTECTION**			
Identification Card	94.4%	90.2%	81.1%

* These data summaries were taken from the Living Standards Measurement Survey World Bank Report Bodewig et al, 2005.

All other data summaries were calculated by the author using PASW 17 ^© ^University of Ottawa 2009

*Early childhood development *influences health later in life both directly, through good nutrition and lifestyle, and indirectly through skills development and education [22]. As can be seen in table 1, Roma have much lower birth weights and experience greater stunting than non-Roma children. In regards to education, 73.6% of Roma children are enrolled in primary school (grade 1-8) with only 27.2% graduating primary school, compared to 76.9% of the general population graduating from primary school [18].

*The daily living conditions and housing *in which people live have a major impact on their health status. As indicated in table 1, a much larger number of persons living in Roma settlements live in housing with inadequate access to clean water, sanitation and electricity.

*Fair employment *refers to safe, secure, and fairly paid work and includes both the conditions and the nature of the work itself. As seen in table 1, a large number of Roma, 31.9%, remain unemployed. Of those Roma that are employed, only 20% have access to full-time employment with benefits. The remainder indicated that they worked seasonal, part-time, or contract jobs. Informal employment conditions may lead to both physical health hazards and health issues related to the stress of not having a steady income.

*Social protection *is important in that it provides a safety net for the poorest and most vulnerable in society. In order to access social protection services in Serbia, one must possess identification documents. As indicated in table 1, it is estimated that 18% of Roma do not possess a health insurance card, compared to 5.6% of the general population. There are currently no systematic legal mechanisms in place to assist the Roma in becoming registered citizens [4].

*Universal health care *and the factors that affect how people interact with the healthcare system can be considered as determinants of health. The acronym PROGRESS can be used to describe the individual equity factors that may affect healthcare usage. PROGRESS stands for place of residence; race, ethnicity, and culture; occupation; gender; religion; education; socioeconomic status; and social capital [6]. Table 2 outlines how access to healthcare, and consequently the realization of the right to health, is affected by PROGRESS.

**Table 2:** How access to healthcare is shaped by PROGRESS

*Place of Residence*:	The Roma tend to live in ghettoized settlements on the outskirts of cities, which are separate from the general population's place of residence. As seen above, slum housing in these settlements is quite common. In addition, the distance and lack of transportation from primary care centers may be an issue for some poor individuals.
*Race/ethnicity/culture*:	The Roma people have been widely discriminated against throughout Europe because of their ethnicity and culture. Decades of social exclusion have created a situation in which healthcare workers are not educated in cultural sensitivity to the Roma population [8].

*Occupation*:	High rates of unemployment amongst the Roma may be the result of a number of issues including lack of education and social exclusion. As many Roma are not formally employed, they do not have access to health insurance under the Health Insurance Fund [9].

*Sex/Gender*:	Roma women and single mothers are particularly vulnerable due to their precarious position and reliance on those with power within the family structure [43]. A systematic review of studies on Roma women in Central and Eastern Europe revealed that Roma women tended to have more issues related to reproductive health, have their first pregnancy earlier, and have less knowledge about contraceptive methods than the general population [44].

*Religion*:	Religion and ethnicity are closely intertwined in Serbia and in many cases it is difficult to identify discriminatory acts as primarily religious or primarily ethnic in origin. The lack of communication between the general population and the Roma people has caused religious tensions in the past around patient preferences and the refusal of treatment. Analyses of patient preferences and values would aid in the cross-cultural translation of interventions.

*Education*:	Education is a major predictor of success in breaking out of the cycle of poverty and ill health. Improvement in the education of public health and prevention among mothers has consistently been linked to the better health status of children.

*Socioeconomic Status (SES)*:	Poverty has consistently been linked to poorer health status. In Serbia, 58% of the Roma are living below the World Bank absolute poverty line, defined as purchasing power parity of USD 4.30 per day, compared to only 9% of the general population [13]. There is no data on chronic poverty in Serbia; however, it is generally acknowledged that a much larger percentage of the Roma live in chronic poverty than their non-Roma counterparts.

*Social Capital*:	Roma appear to be high in social capital as a result of close-knit families and communities. Social networks provide day care for children of ill parents, palliative care to the elderly by younger generations in the household, and care giving to neighbours and friends [17]. Recent plans to move or demolish Roma settlements may have severe detrimental impacts on these social networks and support mechanisms.

Analyzing the themes across which deprivation occurs clearly reveals that inequity may exist across socioeconomic, political, ethnic, and cultural dimensions [21]. Any analysis of the Roma's situation must take into account their precarious position in society if they are to be effective at reducing current inequities.

## 2.0 Methods

### 2.1 Choice of example

The Serbian Millennium Development Goal Progress Report indicates that although there has been a marked reduction in child mortality over the last 10 years, high child mortality and morbidity rates still persist among the Roma [23]. Acute respiratory infection (ARI) is the leading cause of mortality among children under the age of 5 years. The use of antibiotics to treat ARI is considered as the key intervention [18]. ARI was defined in this study as the presence of an acute cough in the previous 2 weeks accompanied by rapid or difficult breathing, and whose symptoms were due to a problem in the chest, or both a problem in the chest and a blocked nose, or whose mother did not know the source of the problem [24].

### 2.2 Framework for analysis

In this section of the paper, we will assess whether the Roma, as a vulnerable population, are able to effectively access primary care services, and if not, what barriers prevent them from doing so. We use the example of acute respiratory infection (ARI) in children under the age of 5 years to demonstrate where the gaps in coverage exist.

**Figure 1:**
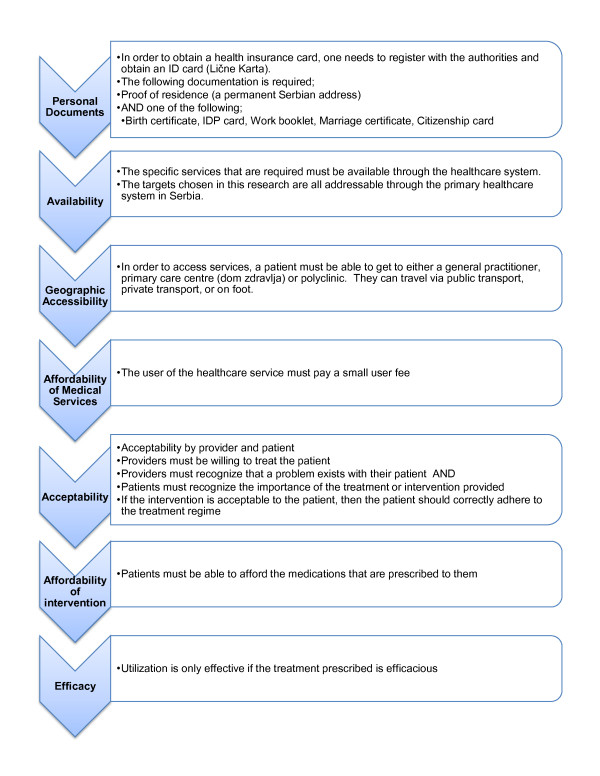
**Steps that must be undertaken by an individual in order to receive effective coverage**.

We use the effective coverage framework to answer the question; "What barriers do the Roma population face when attempting to access primary healthcare services?" Effective coverage is a metric recently proposed by the World Health Organization. At the individual level, effective coverage can be defined "as the probability of receiving a necessary health intervention, conditional on a health care need" [25]. This framework explicitly considers the quality of access, defined as; affordability, availability, accessibility, acceptability and effectiveness of health services [26]. As can be seen in Figure 1, whether a population in need of an intervention actually utilizes that intervention is dependent on a number of health system factors that can drastically reduce the actual proportion of a population benefiting from needed care.

### 2.3 Sources of data

Major Serbian policy papers were analyzed to determine which data source they relied on. The reports searched were the Poverty Reduction Strategy Paper [27], The Government of Serbia MDG Monitoring Framework [23], The Minority Rights Centre Decade of Roma Report [28], Decade Watch [29] and the World Bank's Programmatic Poverty Assessment for Serbia and Montenegro [30]. The references were searched for other reports on Roma health that may contain additional datasets. Subsequently, reports on health in the Roma from international non-governmental organizations were searched. Individual studies on the Roma people in Serbia were searched in PubMed, EMBASE, and Scopus. Seventeen experts, defined as those persons leading change for the Serbian Roma population, both at the government level and at the non-governmental level, were approached and asked whether they were aware of any additional studies that had been conducted on Roma health in Serbia.

In all cases, authors and data holders were contacted in order to request the original datasets. Only data disaggregated by population group were included in the final analysis. Secondary data on each of the factors that affect effective coverage were gathered from three main sources:

• the 2007 World Bank Living Standards Measurement Survey,

• the 2006 UNDP Vulnerability Survey, and

• the 2005 UNICEF Multiple Indicators Cluster Survey.

The datasets were all collected as part of household surveys and the data were stored as SPSS raw data files. The sample size and response rates are reported in table 3 below. The identified datasets were searched for data relevant to the quality of access indicators previously selected.

**Table 3:** Population size and response rates of the secondary data sources analyzed

Data source	Population size	Response Rate
**LSMS 2007** **Living Standards Measurement Study 2007, World Bank**	5557 households 17375 individuals	78% 80.6%

**UNDP 2006** **Vulnerability Report 2006**	1201 households 4582 individuals	Not reported

**UNICEF MICS 2005** **Multiple Indicator Cluster Survey (MICS) 2005**	5557 households 7516 women 3777 children	93% 89.1% 92.0%

### 2.4 Population

Data were disaggregated by population group: the general population (or top 80% of the population in terms of wealth), the poorest 20% (not including the Roma), and the Roma population. The population groups were defined as follows: first the datasets were divided by wealth quintile. Following this, the Roma was separated from the total population group. Finally the predefined poorest quintile was separated from the total population, leaving the general population. The three population groups are distinct and there is no overlap between the poorest quintile, Roma, and the general population.

Effective coverage of the Roma was compared to both the general population and the poorest quintile in order to identify gaps in coverage. By comparing the Roma to the poorest quintile, this research reveals that the Roma's situation is more than merely a result of their lower socioeconomic status and that other factors such as place of residence, occupation and gender also affect the Roma's ability to utilize health services effectively.

The breakdown of persons surveyed is displayed in table 4.

**Table 4:** Description of population groups

		LSMS 2007			MICS			UNDP		
		**General Population**	**Roma**	**Poorest 20%**	**General Population**	**Roma**	**Poorest 20%**	**Non-Roma living nearby to settlement**	**Roma**	**Poorest 20%**

**Gender**										

	Male	48.6%	50.4%	47.7%	48.3%	49.9%	49.1%	77.2%	78.4%	77.1%

	Female	51.4%	49.6%	52.3%	51.7%	50.1%	50.9%	22.8%	21.6%	22.9%

**Urban/Rural**										

	Urban	58.4%	45.8%	35.6%	61.7%	69.4%	14.6%	56.1%	27.7%	52.5%

	Rural	41.6%	54.2%	64.4%	38.3%	30.6%	85.4%	43.9%	72.3%	47.5%

**Age**										

	0-14	13.4%	34.4%	13.4%	22.4%	36.5%	20.5%			

	15-29	20.4%	23.9%	15.2%	19.5%	26.2%	16.3%	8.6%	15.1%	11.0%

	30-49	27.1%	26.5%	23.5%	27.7%	24.0%	23.2%	43.0%	42.8%	48.3%

	50 and over	39.0%	15.1%	48.0%	30.4%	13.3%	40.1%	48.4%	42.1%	40.8%

**Wealth Quintiles**										

	Poorest	NA	65.5%	100%	NA	66.8%	100%	NA	33.2%	100%

	Second	26.6%	21.3%		20.6%	12.2%		24.3%	22.6%	

	Middle	26.5%	9.6%		21.3%	5.5%		24.7%	18.5%	

	Fourth	24.7%	3.5%		21.7%	1.8%		25.1%	14.9%	

	Richest	22.2%	0%		21.7%	0.5%		25.9%	10.7%	

### 2.5 Data Analysis

The three identified datasets were searched for questions related to the use of primary healthcare services. These questions were separated according to themes based on the factors that affect quality of access: accessibility, affordability, acceptability, availability and effectiveness.

The results were analyzed using PASW Statistics 17 (formerly known as SPSS). For each question analyzed, the absolute number and proportion of the population were recorded. The confidence intervals around each proportion were calculated using the normal approximation to the binomial. In instances where the sample sizes were too small, the 95% confidence intervals were calculated using the graph for binomial confidence intervals. A Pearson chi-square test was conducted for each question. In cases where the chi-square test was statistically significant, the post-hoc Bonferroni test for significance was conducted at the 0.05 and 0.01 levels of significance. The absolute and relative differences between the Roma and general population and the Roma and the poorest quintile, as well as the 95% confidence interval around the difference between each population group, were calculated in Microsoft Excel. The measure of association between variables of the same theme, for example the three questions related to accessibility, were calculated using the Pearson's phi for nominal dichotomous data.

Questions that could not be answered using data from the three datasets previously identified were analyzed separately. Data on efficacy, adherence and diagnostic accuracy typically could not be found in the three datasets. In these cases, systematic reviews and large randomized control trials were searched for proxies.

## 3.0 Results

In order to determine whether children in need are accessing the primary care system and thus receiving the correct course of treatment, i.e. antibiotics in this case, we need to analyze the factors that affect the quality of access. The additional file outlines the process that a child must undergo in order to access the primary healthcare system and thus antibiotics [see additional file 1 - Summary of results]. The third column of this table outlines which populations are affected in each case.

**Figure 2:**
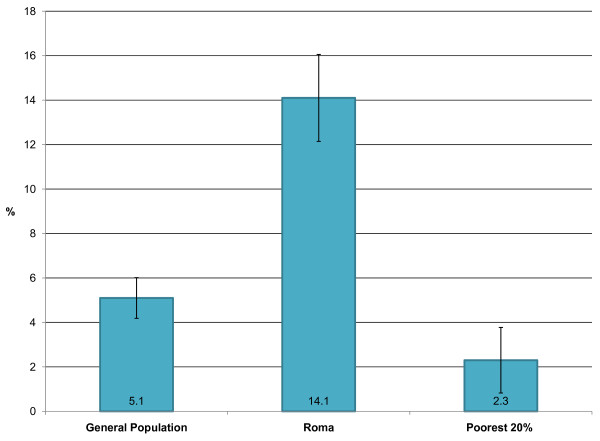
**The proportion children under five with symptoms of acute respiratory infection in previous two weeks**. The 95% confidence intervals for each proportion are indicated on the graph. The Sample sizes are: General Population 2223/Roma 1218/Poorest quintile 397. The data source is the MICS 2005 (UNICEF).

*Need: *Registered need was defined by the number of children less than 5 years who presented with an ARI in the previous 2 weeks. Figure 2 reveals the proportion of children in each population group under the age of five years who had symptoms of an acute respiratory infection within the previous two weeks. The difference between both the Roma and the general population and the Roma and poorest quintile is statistically significant (p < 0.01). Thus we can conclude that need is much greater in the Roma population. This may be due to a number of factors including the fact that the Roma tend to live near industrial areas where the rates of pollution are high.

As can be seen in the summary of results additional file [see additional file 1 - Summary of results], the first step in the process of accessing treatment is that a child must possess the necessary documents; in Serbia this is a health insurance card. Kingston et al [31] have described in detail the difficulty Roma face in accessing healthcare without the necessary documents. In addition, this research has shown that 18.9% (95% confidence interval = 15.3 - 22.5%) of Roma do not have health insurance cards compared to both the general population, 9.8% (95% confidence interval = 8.8 - 10.8%), and poorest quintile, 5.6% (95% confidence interval = 5.2 - 6.0%). This situation is worse among the rural population with 23.7% of the rural Roma population lacking a health insurance card compared to 13.5% of the urban Roma population (p = 0.04). Although large numbers of the Roma do not possess a health insurance card, there is anecdotal evidence that they are in fact receiving treatment by sharing health cards, sharing medications, and receiving treatment from physicians illegally who turn a blind eye to the fact that they are lacking a health card.

*Availability *is measured by whether a child has a family doctor. Only 40.1% (95% confidence interval = 36.7 - 43.5%) of the Roma population have a family doctor but there is no statistically significant difference between any of the population groups.

*Accessibility *refers to whether a child is actually able to present at a primary care clinic or to a general practitioner. Although only 11.6% (95% confidence interval = 9.4 - 13.8%) of the Roma population lived further than 5 km from a primary care centre this is still statistically significantly less (p < 0.01) less than either the general population or poorest quintile. Not surprisingly, the situation is worse among the rural Roma communities with 15.3% of the rural Roma population living greater than 5 km away from any primary care facility compared to 5.9% of the urban Roma population (p < 0.01).

*Affordability *can be broken down into three separate questions: (1) whether health services were not used due to prohibitive costs, (2) whether parents could afford to purchase medications, and (3) whether individuals were willing to pay for services. Although much of the health system is publicly funded, a user fee may be a hindrance to those who are extremely poor. Also not all services are funded under the public system and some services, such as pharmaceuticals, are privately financed [32].

**Figure 3:**
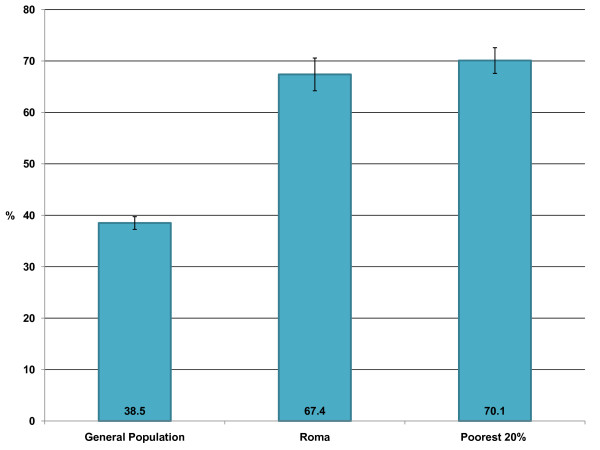
**The proportion of the population that could not afford to purchase prescribed medications within the previous 12 months**. The 95% confidence intervals for each proportion are indicated on the graph. The Sample sizes are: General Population 5961/Roma - 831/Poorest quintile - 1292. The data source is the UNDP Vulnerability Survey 2006

(1) *Were the Roma able to afford health services? *The 2003 Health Policy Document of Serbia mandates that vulnerable persons, including the Roma, do not have to pay the user fee for medical services or medicines, which is otherwise obligatory [33]. On the one hand, data show that there is no difference in either the likelihood of out-of-pocket payments or in the absolute amount that is paid out by the Roma when compared to the general population and poorest quintile; however, looking at whether the Roma actually used services reveals that 56.4% (95% confidence interval 43.3 - 69.5%) of the Roma population did not use healthcare services in the previous month when they were in need due to the fact that the services were deemed too expensive. This is statistically significantly greater than the general population and the poorest quintile (p < 0.01). Thus the Roma are much less likely to be able to afford to pay for health services.

(2) *Were the Roma able to afford medications? *In addition to not being able to pay for health services, it also appears that Roma are less likely to be able to afford medications. Figure 3 describes the proportion of persons that could not afford to buy prescribed medications within the previous 12 months. There is no statistical difference between the Roma and poorest quintile and both are statistically significantly higher than the general population (p < 0.01). This lack of difference between the poorest quintile and the Roma population may be due to the fact that the poorest quintile is more likely to receive social assistance than the Roma population, thus easing the burden of their poverty.

(3) *Were the Roma willing to pay? *Any discussion of ability to pay needs to take into account an individual's willingness to pay, especially when doing an analysis of socioeconomic status [34]. Further research is required into how the Roma value and use services - i.e. is it matter of them not valuing the services and therefore not prioritizing and paying for services, or is it a matter of the cost of the services.

In conclusion, we see that all three population groups are required to pay the same out-of-pocket expenses, despite the fact that it is possible to waive fees for poorer persons. The Roma are much less likely to be able to afford these costs, and possibly also less willing, therefore not utilizing the services.

*Acceptability *was measured as recognition of the importance of the illness. It was assumed that if a mother sought advice from any source, then she saw her child's illness as important. Recognition of the importance of the illness was measured as the proportion of mothers who sought advice from an outside source including: seeking advice from neighbours, holding a religious ceremony, and taking the child to a medical centre. There was no statistically significant difference between the Roma and general population or poorest quintile with regards to recognition of ARI as a serious illness. Therefore, mothers from all population groups recognized ARI as a serious illness.

*Effectiveness *is defined as a combination of efficacy and diagnostic accuracy. Since there is no data on efficacy or diagnostic accuracy within the Roma in Serbia, studies from other sources were sought. A systematic review [35] on the efficacy of antibiotics to treat ARI found that 88% of the treatment group recovered, compared to 66% in the non-treatment placebo group. No studies indicating that there are differences across population groups could be found. Studies estimating diagnostic accuracy are limited across aspects of equity; therefore only the general population is compared to the Roma. Diagnostic accuracy in the general population is estimated at 73%. Only one study could be found on diagnostic accuracy in children of disadvantaged populations. This study compared children of different ethnic backgrounds in the United States. From this study, it appears that children from more economically disadvantaged households are less likely to be accurately diagnosed by a healthcare professional than their richer counterparts^1 ^[36].

### Actual Utilization and Effective Coverage

**Figure 4:**
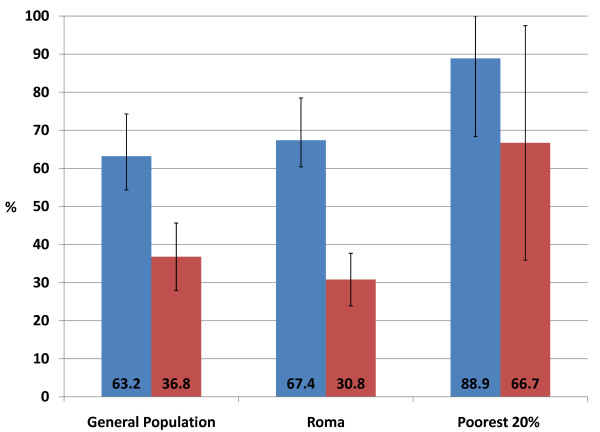
**The proportion of children under 5 with a suspected ARI that received any medication versus antibiotics**. Blue bar = Actual utilization - the child was given any medication to treat the acute respiratory infection. Red bar = Effective coverage - the child was given the correct treatment (in this case antibiotics) to treat the acute respiratory infection. The 95% confidence intervals for each proportion are indicated on the graph. The Sample sizes are: General Population - 114/Roma - 172/Poorest - 9. The data source is the MICS 2005 (UNICEF).

Actual utilization is defined as the number of persons who receive an intervention regardless of whether this is the correct intervention, or one step towards the final intervention. In this scenario, actual utilization is defined as the proportion of children with an ARI who received *any *medication to treat this condition. As can be seen in Figure 4, children from all three population groups were equally likely to receive some form of treatment for the ARI. Actual utilization is distinct from effective coverage. Effective coverage is defined as the proportion of children with an ARI that received the correct treatment for this condition, defined in this example by the UNICEF MICS survey as the prescription of antibiotic medication. It is important to note that effective coverage can only be measured using clinical outcomes, in this case a lab test confirming the presence of ARI before antibiotics are prescribed and again after the treatment course confirming that the ARI has been resolved. Thus treatment with antibiotics is used as a proxy for effective coverage in this application. As can be seen in Figure 4, there was no statistically significant difference between the proportion of children that receive the correct treatment in any of the population groups. Thus children from all three population groups were equally likely to receive the correct treatment for ARI. Hence effective coverage is equal across all three population groups.

If effective coverage is analyzed in relation to actual utilization, as presented in Figure 4, we see that there is a large gap between the treatment that is administered and the treatment that is actually required. Thus although children under five years of age in all three population groups are receiving treatment, the treatment is not necessarily an effective treatment for the condition. There is however no statistically significant difference across the three population groups for either effective coverage or actual utilization. Some may argue that if effective coverage and utilization are not statistically significantly different, then the outcome is that all population groups have equal access to health services. In reality, however, the Roma people may be in an even more precarious position if they are circumventing the health system. For example they may be receiving treatment illegally, they may be borrowing health cards from friends and relatives, or they may be sharing medications. Each of these cases could lead to unpleasant outcomes. Physicians may choose to stop treating them or ask for bribes or gifts. Sharing health cards among children has resulted in anecdotes of children being 'dead' in the eyes of the law when the child borrowing the health card has died in hospital. Or sharing medications may lead to incomplete courses of antibiotics or using the wrong medications to treat illnesses. Therefore, further research into how the barriers to access are being overcome should be conducted in order to determine how the Roma are accessing health services and why effective coverage and utilization show no statistically significant difference across population groups when the differences in the quality of access is so clear.

To summarize: Roma children are significantly more likely to experience an ARI than either the general population or the poorest quintile. All three population groups are equally likely to not receive the correct treatment regime of antibiotics. Thus there is a considerable gap between actual utilization and effective coverage for all three population groups. An analysis of the factors that affect quality of access reveal that personal documentation is a statistically significant problem; availability is not an issue that disproportionately affects the Roma; however the geographical accessibility and affordability are substantive issues that disproportionately affect the Roma population. Affordability of services affected the Roma and the poorest quintile and affordability of medications significantly affected all three population groups. With regards to acceptability, mothers from all three population groups are equally likely to recognize the importance of seeking treatment. Looking at effectiveness, Roma and poor children may be less likely to be accurately diagnosed, but once diagnosed may be equally likely to benefit from the treatment.

Areas for improvement with regards to ARI are both general and specific. In general terms, the Roma should be assisted in applying for personal documentation, the geographical accessibility of clinics needs to be addressed, and the costs of healthcare visits and medications should be reviewed. Areas for improvement specific to ARI are the costs of antibiotics and the diagnostic accuracy of providers. In addition, research on why a larger proportion of Roma are in need is required.

## 4. Discussion

### Limitations

Five potential limitations of this study have been identified: self identification of Roma, lack of disaggregated data, data collection by different organizations, use of secondary data, and limitations of the effective coverage framework.

In any study of a marginalized population group, self identification raises the problem that members of the excluded group may not identify themselves as part of that group due to discrimination. On the other hand, 'outsider' definitions of ethnicity may reflect stereotypes or cause further discrimination. This study attempted to overcome this issue by defining the population based on their place of residence, i.e. those persons living in Roma settlements. By including only individuals that live in settlements, it is possible that important Roma population groups are excluded. In addition, non-Roma living in these areas are by definition included. It is therefore important to note that these results are only generalizable to those persons that live in Roma settlements and not to the Roma population as a whole.

A second potential limitation of this study is the fact that data in Serbia are not usually disaggregated at the population level and so although numerous sources were searched, only three datasets were included. This meant that although this study was able to report on some aspects of effective coverage, such as affordability, data on other factors such as discrimination and patient satisfaction were not available at the disaggregated level.

The third potential limitation of this study is the fact that data were drawn from a number of different sources. Although this was necessary in order to incorporate as much data as possible into the study, it is important that while reading this study, one takes into account the varying populations surveyed as detailed in table 3.

The fourth potential limitation is with regards to the use of secondary data. Secondary data are only as good as the research that produced them and thus may have limitations. Although this research relies on the documentation of the original datasets, there may have been bias in the sample collection phase. Given the description of survey methodologies as well as discussions with individuals in Serbia who administered the surveys, the data has been deemed as both valid and reliable. In addition, the surveys were all conducted in Serbian and then translated to English; therefore there may be jargon in the wording that is not captured in the translations. However, following discussions with data holders in Serbia, it appears that the concepts were accurately interpreted and are reflected in the English datasets utilized in this research.

The fifth limitation is a limitation of the effective coverage framework in general. Effective coverage can only truly be measured through clinical confirmation. For example, in the acute respiratory infection (ARI) application, use of antibiotics was used as a proxy for effective coverage, but true effective coverage can only be measured by clinical assessment and laboratory investigation. In this case a clinical assessment, laboratory test, or x-ray confirming the presence of ARI before antibiotics are prescribed and again after the treatment course confirming that the ARI has been resolved. As clinical confirmation is difficult to gather on a large scale, application of this framework usually requires that proxies be used to measure the extent of coverage.

### Discussion of Policy Implications

A summary of the policy recommendations that follow from the research is provided in table 5. The recommendations outlined by this research were formulated using the Global Equity Gauge framework. According to this framework in order to turn research into viable policy recommendations, one needs to take into account the context, analyze which subgroups are affected, determine what is currently being done in that situation, and what has worked in similar situations. Finally based on all this information policy recommendations and recommendations for future research can be offered.

**Table 5:** Policy Recommendations

**Personal Documents and Registration**

1. Until such a time as political will to legalize informal settlements exists, the current settlements should be equipped with temporary house numbers. In addition households should register with the local authorities to confirm their residence status.

2. An integrated strategy at the national level that allows Roma to register their permanent address as a local community centre needs to be implemented as an interim solution.

3. Standardization and training to guide administrators on when to reduce fees would help maintain consistency and minimize discrimination.

4. Review the registration procedure in order to determine where the process is arduous and implement administrative processes that overcome these barriers. For example, representatives within the settlements could be hired to assist in Roma the completion of necessary forms as well as educate on the application process.

5. An evaluation of the Roma health mediator program should be conducted in order to determine whether the program is working. This should be completed in conjunction with a publication of best practices from the evaluation.

6. The number of unregistered persons needs to be determined so that registration processes undertaken by the UNHCR and Praxis may be monitored as they continue to persevere with the registration of chronically unregistered Roma.

**Availability of Physicians**

7. Although the availability of physicians is not an issue that disproportionately affects the Roma, research into the root causes of why persons do not have a chosen practitioner should be undertaken. With this knowledge, an integrative plan that takes into account the recommendations from the 2006 World Health Report and Global Health Workforce Alliance should be developed.

**Geographical Accessibility**

8. Geographical accessibility for rural Roma should be made a priority and evaluation of the feasibility of identified interventions would be helpful within the Serbian context.

**Affordability**

9. Out-of-pocket payments for both services and medications should be reduced or eliminated as rapidly as possible.

**Discrimination**

10. A comprehensive sensitivity training program aimed at all levels of health workers needs to be implemented; this includes training in the medical and nursing schools as well as sensitivity training in the workplace. In addition, internships in Roma settlements for medical and nursing students may improve relations.

11. The continued assistance to individual Roma persons to help realize their rights is important as this creates a culture of empowerment.

12. Public campaigns educating Roma on their rights, including the right to healthcare need to be implemented as a priority.

This exercise was undertaken for each of the policy recommendations outlined below. These are organized under several headings, which reflect the findings of the research: personal documents, availability of medical personnel and services, affordability of services and medications, geographical accessibility to clinics, and discrimination. It should be noted that these recommendations address only the specific findings of this study, and not the overall performance of Serbia's health system with respect to the right to health care.

In a study that used 54 indicators to assess the performance of 194 countries' health systems with respect to the right to health, Backman et al [37] identified acquisition of personal documents through a civil registration system as a necessary precondition to the realization of the right to health. This observation is borne out by the work reported here, which found lack of a health insurance card to be a substantial barrier to accessing care. We found that the reason that Roma do not possess these documents has to do less with knowledge on how or where to register than with actual characteristics of the registration process. Four main barriers are involved: the lack of a permanent address (hence policy recommendations 1 and 2), financial barriers (policy recommendation 3), procedural barriers (policy recommendation 4 and 5), and chronic non-registration (policy recommendation 6).

Much evidence shows the importance of family physicians for access to health care [7]. Our analysis of the availability of services, measured using access to a family physician as a proxy, revealed that availability is constant across all population groups. Thus, although the availability of a family physician could be increased for the entire population, it is not a problem that is specific to the Roma population. Backman et al [37] indicate that adequate health workforce planning is necessary to the realization of health as a human right. Policy recommendation number 7 is based on the 2006 World Health Report on Working Together for Health, which outlines a number of strategies for scaling up the workforce including recommendations for training new health workers, optimizing the current workforce members' skills, and managing the migration of health workers [38].

Geographical accessibility to primary care centres is high, with 88.4% of the total Roma population living within 5 km of a primary care centre. Although this level of access is excellent, rural Roma are still much further away from primary care centres than their urban counterparts with 23.7% living further than 5 km from a primary care centre (p = 0.04). Although coverage of the population in Serbia is excellent, it is the rural population that is most disadvantaged. A systematic review [39] on increasing the proportion of health professionals practicing in rural and underserved areas found very low quality evidence to support the following interventions;

- Health professionals from rural backgrounds are more likely to practice in rural areas,

- Evidence from 4 quasi-randomized trials suggests that clinical rotations in a rural setting may influence a small proportion of medical students subsequent decisions to work in an underserved area,

- The effectiveness of compulsory placement has been assessed by descriptive surveys with inconclusive results,

- Loan repayments, direct incentives and medical resident-support programs to encourage rural placement have the highest service completion rates and physician retention rates.

Although these interventions are supported by very low quality evidence, this is the best evidence that exists and further research into the viability of these interventions should be conducted in the Serbian context if they are to be implemented. The analysis by Backman et al. [37] attaches special importance to measures to ensure adequate access to health care for rural populations, but evidence on how to achieve this is incomplete [39], hence, the need for further research as part of the process of health workforce planning (recommendation 8). The Government of Serbia is already training and placing a network of Roma health mediators in both rural and urban areas. It is hoped that these persons could act as inter mediatory between the Roma and healthcare workers. The goal of the Roma health mediators is also to identify community health problems and work with health professionals to aid them in visiting and performing clinics in the community.

The analysis of the affordability of services revealed that all three population groups are required to pay the same out-of-pocket expenses, however the Roma are much more likely not to be able to afford these costs and therefore not utilize the services; 56.4% of the Roma population did not utilize services in the previous month because they were too expensive. Increasing the price of health services tends to decrease demand, regardless of true need [40], and implementing co- payments for drugs results in a decrease in medication usage for life sustaining drugs and chronic conditions [41]. Hence the urgency in reducing or eliminating user fees and co-payments (recommendation 9).

Finally, it would appear that discrimination against the Roma within Serbia as a whole remains a serious problem; a November, 2010 European Commission report progress toward European Union standard as a potential candidate for EU membership concluded that: "The majority of the Roma population ... continues to face discrimination in particular as regards access to education, social protection, health care, employment and adequate housing" [42]. Strategies to eliminate discrimination have in the past focused on three main areas: (a) the adoption of anti-discrimination laws, (b) training for healthcare personnel (recommendation 10), and (c) realization of rights for Roma people (recommendation 11 and 12). Participation of Serbia in the European Decade of Roma Inclusion provides a basis for cautious optimism about the future of these recommendations, despite the November 2010 findings.

## 5. Conclusions

This report is timely as recognition of the importance of measuring and implementing equitable programs for the Roma population in Serbia has become a priority in the last three years. Specifically, the Government of Serbia has not only demonstrated an internal commitment to its people, through the adoption of a set of Health Related Laws mandating that services be physically, economically and geographically accessible^1^, but has also demonstrated a commitment at the international level through participation in the European Decade of Roma Inclusion. It is hoped that this research has added to the knowledge and discussions around equity in access to healthcare services for the Roma, and has supported the urgent need for implementation of important public policy recommendations.

## Competing interests

The authors declare that they have no competing interests.

## Authors' contributions

LI, OA, JP and PT contributed to the conception and design of the study. LI and OA acquired the data. LI conducted the data analysis with assistance from JP and PT. LI, OA, JP and PT interpreted the results. All authors were involved in the drafting and revising of the manuscript. All authors read and approved the final manuscript.

## Pre-publication history

The pre-publication history for this paper can be accessed here:

http://www.biomedcentral.com/1472-698X/11/10/prepub

## Supplementary Material

Additional file 1 **Summary of process and results**. This table outlines the process which a child must go through before they can be said to have effective coverage. In addition, the results of our study are summarized in the last column of this figure. A table including the process of moving through the health system and descriptive results of the data analysis. Click here for file
