# Supplementary material for: Access to primary healthcare services for the Roma population in Serbia: a secondary data analysis
Source: BMC Int Health Hum Rights. 2011 Aug 18;11:10. doi: 10.1186/1472-698X-11-10 (PMC3175440; doi:10.1186/1472-698X-11-10)
Supplement: Additional file 1 — Summary of process and results. This table outlines the process which a child must go through before they can be said to have effective coverage. In addition, the results of our study are summarized in the last column of this figure. A table including the process of moving through the health system and descriptive results of the data analysis. [file 1472-698X-11-10-S1.PDF]

	Process to Access Primary Healthcare Services	Population Affected
**Need**	The number of children less than 5 years who presented with an ARI in the previous 2 weeks	**Total Population**: The occurrence of acute respiratory infection is quite high across the general population, Roma and poorest quintile.<br><br>**The Roma:** Roma children are much more likely to present with symptoms of acute respiratory infection than either the general population or poorest quintile.
**Personal Documents**	In order to obtain a health insurance card, one needs to register with the authorities and obtain an ID card (Lične Karta).<br><br>The following documentation is required;<br>- Proof of residence (a permanent Serbian address)<br><br>AND one of the following;<br>- Birth certificate<br>- IDP card<br>- Work booklet<br>- Marriage certificate<br>- Citizenship card	**Total Population**: Access to personal documents is adequate across the general population and poorest quintile.<br><br>**The Roma:** The Roma are less likely to have personal documents.<br><br>**Subgroups of Roma**: Within the Roma population, rural dwellers are worse off.
**Availability**	It is assumed that a family doctor will have access to the knowledge, diagnostic tests, and equipment that are required to diagnose and treat ARI.<br><br>Therefore the proxy for availability is whether a child has a family doctor.	**Total Population**: The proportion of population with a family physician is low for the general population, poorest quintile and Roma.<br><br>**The Roma**: The Roma are not disproportionately affected.<br><br>**Subgroups of Roma**: Within the Roma population, the following groups are less likely to have a family physician:<br>- males,<br>- urban dwellers,<br>- those with incomplete high school, and<br>- the employed.
**Geographic Accessibility**	In order for an ARI to be treated by a physician, the child must present to either a general practitioner or a primary care centre (dom zdravlja).<br><br>They can travel via public transport, private transport, or on foot.	**Total Population**: Coverage is excellent with a large majority of the general population, poorest quintile, and Roma living within 5km of a primary care centre or family physician<br><br>**The Roma**: The Roma are still disproportionately affected with more Roma living further than 5km from a primary care centre, family physician or polyclinic.<br><br>**Subgroups of Roma**: Within the Roma population, the following groups are less likely to be further than 5km from a primary care centre (dom zdravlja):<br>- the rural population<br>- the unemployed
**Affordability**	**Affordability of Medical Services:**<br>- The parents of children must pay a small user fee<br><br>**Affordability of Medications:**<br>- Parents must be able to afford to purchase the antibiotics for their ill children	**Affordability of Medical Services:**<br>**Total Population**: most of the general population, poorest quintile, and Roma are able to afford health services.<br><br>**The Roma**: The Roma are disproportionally affected with more Roma not being able to afford health services<br><br>**Subgroups of Roma**: Within the Roma population, all Roma are affected equally.<br><br>**Affordability of Medications:**<br>**Total Population**: The number of persons that can afford to purchase medications is very low among the general population, poorest quintile and Roma.<br><br>**The Roma**: The Roma and poorest quintile are disproportionately affected by the cost of medications.<br><br>**Subgroups of Roma**: Within the Roma population, the following groups are less likely to be able to purchase medications:<br>- those who have not completed high school, and<br>- the unemployed.

**Acceptability**	**<u>Acceptability to Provider:</u>**<br>- Providers should not discriminate against Roma children.<br><br>**<u>Acceptability to Patient</u>**<br>- Parents must recognize that ARI is a serious infection that they should seek treatment for.<br><br>- Parents must find the treatment culturally acceptable.	**<u>Acceptability to Provider:</u>**<br>**Total Population**: No quantitative data currently exists on the extent of discrimination within the health system<br><br>**The Roma**: From case studies, the Roma appear to be disproportionately discriminated against.<br><br>**Subgroups of Roma**: Within the Roma population, there is no data on whether certain subgroups are more discriminated against.<br><br>**<u>Acceptability to Patient</u>**<br>There is no statistical difference between the three population groups with regards to whether parents sought any form of treatment including herbal teas, holding a religious ceremony, and consulting a family member or friend.<br><br>There is no data on any population group regarding the acceptability of antibiotics as a treatment for ARI.
**Effectiveness**	- **<u>Diagnostic Accuracy</u>**: Providers must correctly diagnose a child with ARI and only prescribe antibiotics for bacterial infections<br><br>- **<u>Efficacy</u>**: The antibiotics should show high rates of efficacy in treatment of ARI<br><br>- **<u>Adherence</u>**: Children should adhere to the full course of antibiotics prescribed	**The Roma**: There are limited studies however it appears that efficacy is the same across all three population groups, however diagnostic accuracy is lower in the Roma population.

EFFECTIVE COVERAGE
